# Remembering the romantic past: Autobiographical memory functions and romantic relationship quality

**DOI:** 10.1371/journal.pone.0251004

**Published:** 2021-05-03

**Authors:** Cagla Aydin, Asuman Buyukcan-Tetik

**Affiliations:** 1 Psychology Program, Faculty of Arts and Social Sciences, Sabanci University, Istanbul, Turkey; 2 Department of Psychology, Norwegian University of Science and Technology, Trondheim, Norway; Technion Israel Institute of Technology, ISRAEL

## Abstract

Do the reasons why we think about our memories and share them with others have implications for our romantic relationship quality? In the present series of studies (total *N* = 1,102), we aimed to answer this question by examining whether the self (e.g., creating a stable self-image), social (e.g., connecting with others) and directive (e.g., guiding future behavior) functions of regular memories (Study 1, Study 2) and relationship memories (Study 2, Study 3) were related to intimacy and satisfaction in the current relationship. We further investigated these links when relationship memories were shared with the romantic partner (Study 3). Results showed no association between the self-reported uses of memory for regular events and relationship quality. In contrast, the social function served by the relationship events was positively associated, and the directive function was negatively associated with intimacy and relationship satisfaction. When the memories were to be shared with the partner, only social function was related, positively, to the relationship satisfaction. Findings were discussed in terms of the importance of considering the self-reported reasons for recalling an event and understanding of the contextual factors in remembering.

## Introduction

We remember our personal past for many reasons. According to an influential approach, reasons for autobiographical remembering are categorized into three broad functional categories [1]. *Self function* refers to recalling events to maintain a sense that one is the same person over time and keep a positive image of self [2, 3]. Memories are also recalled and shared interpersonally to form and strengthen social bonds or deepen intimacy with others, which is referred to as the *social function* [4]. Finally, the *directive function* refers to using the personal past as a prescription to guide future behaviors as well as problem-solving [5].

A major tenet of the functional approach is that past experiences are adaptively (re)constructed in order to make them meaningful for responding to ongoing changes in one’s ecological context [6]. One such immediate context is one’s romantic relationships where remembering may be consequential in terms of the degree of intimacy we feel toward our partner or the satisfaction we get from the relationship. Therefore, in the present research program, we considered the extent to which the self-reported functions of autobiographical memory relate to two main indicators of one’s romantic relationship quality, namely intimacy and relationship satisfaction in individuals who are currently involved in a romantic relationship.

Prior research has indeed linked different memory functions with romantic relationship quality. Firstly, a strong association is shown between relationship satisfaction and social function. This was not surprising given that social function is defined as recalling past life to be intimate with others [7, 8] or as coordinating the individual histories of the two partners [9]. For instance, Alea and Bluck [7] targeted a form of social function, intimacy function, served by autobiographical memories, and showed that simply the act of recalling (personal) relationship events, in contrast to (impersonal) fictional vignettes, fostered intimacy in long-term romantic relationships. In a similar vein, Alea and Vick [10] reported that frequently rehearsed relationship-defining memories; that is, memories that are used for maintaining close bonds, predicted marital satisfaction.

In terms of self function, the evidence is relatively indirect. It was reported that the use of autobiographical memories for self-related reasons was positively related to having good relations with friends and significant others [11]. Furthermore, given the findings that self function has strong relations with self-esteem [11, 12], and relationship satisfaction and self-esteem are positively associated [13], it is likely that the use of self function would result in increased satisfaction in close relationships. Similarly, based on the evidence that sharing self-relevant information helps develop relationship intimacy [14], a positive association between remembering for self-related reasons and intimacy between partners can possibly be formed.

Finally, a positive association is also reported between the use of directive function and relationship satisfaction. Philippe, Koestner, and Lekes [15] showed that couple-related memories, by way of satisfying psychological needs, such as autonomy, relatedness and competence, actively direct relationship satisfaction. It is worth noting here that Philippe et al. used the term ‘directive function’ rather loosely- to refer to a directive influence on one’s thoughts and behaviors rather than a memory to be instantly used to guide through a conflict. Based on prior arguments that directive function helps individuals navigate difficult emotional situations [5, 16], an increase in the frequency of the use of memories for directive purposes, for instance, problem-solving, may indicate a tendency to prevent or resolve relationship conflicts in a constructive manner. This, in turn, may benefit relationship quality.

All in all, the reported positive associations suggest that using autobiographical memories functionally benefits the romantic relationship experience. So far, the links from memory functions to relationship quality have been formed by unpacking singular functions. While it is worthwhile to identify individual functions for a fine-grained analysis on how memories function in social context [8], a global analysis of functions is also critical to assess the extent to which functions served by autobiographical memories -relative to each other- are implicated in the dynamic interpersonal sphere. It has been hypothesized that individual memories may serve more than one function depending on the current psychosocial needs of the individual [5, 6]. Therefore, it is possible that uses of memories other than the one targeted in a particular study inadvertently influence the relationship outcomes. A global assessment of the three functions in tandem would allow for examining the relative contributions of each function to romantic relationship quality. It also has ‘heuristic utility’ [17] in broadly thinking about the functions in the close relationship context. Thus, the main goal of present study is to examine how different functions of autobiographical memories relate to the quality of romantic relationships.

Global assessments of the functions of autobiographical remembering have been made possible by using psychometric scales, such as the TALE (Thinking About Life Experiences Questionnaire; [4, 18]) and the RFS (Reminiscence Functions Scale; [19]). Rather than focusing on specific relationship events, these scales focus on recalling over one’s life. For the present purposes, there are several advantages of using a scale of this sort. First, when the focus is on particular events, it is not clear whether functions will generalize across all types of events. For instance, it has been reported that different classes of memories, such as, positive and negative memories, fulfil different functions; contributing to a self-concept and social bonding, and help avoiding dangers, respectively [20]. Second, in previous work on relational outcomes, how memories are used by individuals are largely inferred either by asking individuals to remember intimate relationship events so that the use of the social function is implicated or content analyzing the conversational narratives to identify functional themes [e.g., 17]. Alternatively, the items in the TALE are supposed to tap deliberate uses of the past [8, 18] via self-reported intentions and goals [21]. By explicitly asking participants to think back, and report the usefulness of their own memories, we aim to qualify the connection between conscious recollection of the past and perceived romantic relationship quality.

### Overview of the present studies

In the present series of studies, we examined the association between people’s use of autobiographical memory to serve self, social and directive functions and the quality of one’s romantic relationship. Based on the argument that memories can serve more than just one function [5], this approach would allow for observing the relative contributions of the three conceptually distinct functions to relationship quality. To do so, we relied on individuals’ evaluations of the functions of their own memories by using the TALE scale rather than their responses to specific recollections. Participants had been romantically involved with someone for a minimum of three months, and they were over 18 years of age. First, in Study 1, we started by investigating whether the way individuals use their -everyday- memories are related to the quality of their romantic relationships. Given the finding that all three functions are related to psychological well-being [11], we pursued the question whether the well-being of the relationships depended on the functions one’s personal memories serve. In Study 2, relying on the possibility that relational events may afford potentially different uses compared to everyday personal memories [22], our focus was on how relationship-related memories are used functionally and how those uses were associated with relationship quality. To that effect, we slightly modified the wording in the TALE items to reflect that participants need to think over their romantic life with their current partner when responding to the uses of their memories. Finally, drawing on the possibility that uses of recalling an autobiographical memory may shift when one reports past experiences to a certain addressee, in particular, to the romantic other, we explored whether functions of romantic memories changed when reporting to the current romantic partner and how that related to one’s relationship quality (Study 3).

We operationalize romantic relationship quality as one’s subjective and global evaluation of the relationship [23]. Previous research emphasized the importance of using different relational qualities (e.g., satisfaction, intimacy, commitment, conflict, ambivalence, love) to have a nuanced understanding of how each quality affects the outcomes [e.g., 24–26]. For reasons of brevity, we did not use the whole range of the possible qualities.

Finally, because, to our knowledge, this is the first systematic study to examine the scope of all three functions of memory and their relations to relationship quality, we included an important relational construct and an individual difference variable, attachment style, as a control variable. Attachment style is known to be a strong predictor of relationship quality [27, 28]. Attachment orientations were also reported to be systematically related to what individuals recall about relationship events [29]. Since responses to the items in the TALE can also be thought of “stylistic differences of reminiscence across individuals” [8], controlling for a potentially confounding individual difference variable was critical for our purposes. Attachment orientations have been shown to be associated with memory usage. Whereas attachment avoidance was not related to any of the functions, higher attachment anxiety was found to be related with the social function [30]. How individuals with different attachment styles respond to a break up was found to be mediated by remembering relationship related events [31].

Age and gender were included as the other control variables. Age was included as a control variable because, in the functional remembering literature, it has been shown to be an important factor [e.g., 30]. Gender was included because previous studies on relationship memories point to slight gender differences [e.g., 29].

## Study 1

In Study 1, we establish how functions of generalized memories of life events are associated with romantic relationship quality. Previously, it has been shown that individuals who use their specific memories to serve all three functions reported higher levels of psychological well-being [11, but 32]. Since having intimate romantic relationships are fundamental to socio-emotional well-being [33], we expected that reasons to remember personal experiences would have similar associations with relationship well-being. Even though we did not have predictions as to the relative weights of each function, we predict that social function would be associated with the relationship outcomes to a greater level than the other functions. This is due to the fact that social use of the memory entails maintaining intimacy which should reflect on the quality of our relationships—regardless of the event’s theme (relationship related or not). In fact, Waters [11] study showed that for the use of memories for the recurring events was highly associated with having positive relationships.

## Materials and methods

### Participants

Participant recruitment was conducted through an online crowdsourcing company, Prolific. Inclusion criteria for this study were: being involved in a romantic relationship currently at least for 3 months, being older than 18 and younger than 70, and being a native speaker of English. In the original data, there were 252 entries. Fifteen participants were excluded due to multiple entries, not meeting the inclusion criteria (e.g., being age 18 or over, being in an on-going relationship at least for three months, speaking English as their native language) or not providing the Prolific identity number. Detailed information about excluded participants is available upon request. Sample characteristics of the remaining 237 participants are presented in Table 1.

**Table 1:** Sample characteristics across studies.

	Study 1 (n = 237)		Study 2 (n = 410)		Study 3 (n = 455)	
	*M or %*	*SD*	*M or %*	*SD*	*M or %*	*SD*
**Age**	41.96	10.74	35.97	10.90	36.53	11.48
**Gender (Female %)**	78.10		74.70		70.40	
**Ethnicity (Caucasian %)**	93.20		91.00		90.50	
**Sexual orientation (Heterosexual %)**	95.80		96.00		97.40	
**Relationship type (Married %)**	97.00		58.50		57.30	
**Relationship duration in years**	16.27	10.53	11.35	9.31	12.29	9.86
**Parents (%)**	71.30		56.80		56.30	
**Number of children**	1.99	0.83	1.90	0.88	1.93	0.93
**Education (Bachelor’s %)**	37.10		45.90		33.20	
**Education (High school/GED %)**	38.00		36.80		42.00	

We computed our sample size for multiple regression analyses based on the total number of predictors with the control variables (i.e., 9–14 variables) across our studies. We had an expectation of a medium effect size [34]. Using a desired statistical power level of .8 and a probability level of .05, minimum required sample size was between 113 and 135 across our studies [35], which were all exceeded (*n*_Study-1_ = 237, *n*_Study-2_ = 410, *n*_Study-3_ = 455).

We received ethical approval for this study from Sabanci University Research Ethics Council with the protocol number FASS-2019-49. All participants gave their electronic informed consent for participation before they filled in the survey in return of GBP 1.35.

### Procedure and measures

Questionnaires were administered online. After completing the consent form and reading the instructions, the participants first completed the Thinking about Life Experiences Questionnaire [TALE; 18], and then the relationship intimacy (Inclusion of the Other in Self; IOS; [36]) and satisfaction (Relationship Assessment Scale by Hendrick [37]) scales. The questionnaire ended with the Experiences in Close Relationships-Revised Questionnaire [28] in order to measure attachment orientations and the demographic questions. The TALE was administered before the relationship quality measures; the order was not counterbalanced. Other scales administered but not used for the present work are not reported here (see S1 Appendix).

#### Functions of autobiographical memory

Thinking About Life Experiences Questionnaire [TALE; 18] was used to measure the three functions of memory: self function, social function, and directive function. We asked why participants think back or talk about their life. Sample reasons to assess each function were “when I want to feel that I am the same person that I was before”, “when I hope to also learn more about another person’s life”, and “when I believe that thinking about the past can help guide my future”, respectively. Additionally, there were two items two assess the baseline level (i.e., “In general, how often do you think back over your life?” and “In general, how often do you talk to others about what’s happened in your life?”). A 5-point Likert scale (1 = “almost never”, 5 = “very frequently”) was administered.

We first conducted an exploratory factor analysis using varimax rotation and maximum likelihood estimation to investigate the factorial structure of the TALE in our data. Results revealed three factors with an eigenvalue higher than 1, which altogether explained 52.12% of the variance. All items except one were clearly loading to their function in the original scale. The item “when I want to remember something that someone else said or did that might help me now” had similar loadings on both social function and directive function (loadings of .38 and .34, respectively). Thus, we conducted all analyses first including, and then excluding this item. Each time, we got the same results in terms of the memory functions’ associations to the relationship quality indicators. In the reported analyses, we included this item under the directive function considering the original scale. Besides, exclusion of this item did not increase the internal reliability of the subscale for directive function. For this particular study, the five-item scales for self function, social function, and directive functions had good internal reliability; Cronbach alpha levels of .82, .81, and .84, respectively.

#### Relationship quality

To assess relationship quality, we used two different indicators: intimacy and relationship satisfaction. Intimacy in the participants’ romantic relationship was assessed using the Inclusion of Other in the Self Scale [IOS; 36]. This assessment is done via a 7-point Likert type item composed of seven pictures (degrees of interlocking or isolated circles) representing different levels of closeness. The participants were asked to choose the option that best portrays their relationship. Higher scores (i.e., increasingly overlapping circles) indicated higher levels of closeness. Comparison of different closeness measures showed that the IOS scale “*is a psychologically meaningful and highly reliable measure of the subjective closeness of relationships*” [38, p. 1]. Relationship satisfaction was assessed with six items of the 7-item Relationship Assessment Scale, which was developed by Hendrick [37]. Sample item was “In general, how satisfied are you with your relationship?” One item from the original scale “How much do you love your partner?” was mistakenly omitted in the online version. We administered a 5-point Likert scale (1 = “very low”, 5 = “very high”). Cronbach alpha level was .94 for this study.

#### Attachment

Attachment styles were assessed using the Experiences in Close Relationships-Revised Questionnaire [28]. Each subscale for assessing anxious and avoidant attachment styles had 18 items. Sample items were “I often worry that my partner doesn’t really love me.” and “I find it difficult to allow myself to depend on romantic partners.” for anxious and avoidant attachment styles, respectively. For all items, we administered a 5-point Likert scale (1 = “strongly disagree”, 5 = “strongly agree”). Cronbach alpha level was .94 for anxious attachment and .96 for avoidant attachment.

## Results and discussion

### Descriptive statistics and correlations

Descriptive statistics of and correlations among study variables are presented in Table 2. Correlations revealed that the three functions of autobiographical memory (i.e., self, social, and directive functions) had moderate positive associations with each other. Out of the three functions, only the self function was significantly, but negatively, associated with intimacy and relationship satisfaction. Self function had positive associations with both anxious and avoidant attachment styles. Anxious attachment was positively related to directive function as well.

**Table 2:** Descriptive statistics and correlations among the study variables in Study 1.

Variable		*M*	*SD*	1	2	3	4	5	6
1	**Self function**	2.76	0.83	-					
2	**Social function**	2.91	0.79	**.41**	-				
3	**Directive function**	3.27	0.76	**.53**	**.59**	-			
4	**Intimacy**	5.34	1.59	**-.20**	.00	-.08	-		
5	**Relationship satisfaction**	3.98	0.94	**-.21**	-.02	-.06	**.78**	-	
6	**Anxious attachment**	2.29	0.82	**.25**	.11	**.16**	**-.52**	**-.57**	-
7	**Avoidant attachment**	2.09	0.78	**.21**	-.02	.01	**-.65**	**-.70**	**.64**

*Note*. All values in bold had a p-value lower than .05.

#### Regression results

We regressed intimacy and relationship satisfaction onto self, social, and directive functions of autobiographical memory. We also controlled for the effects of age, gender, attachment styles, and baseline levels of thinking and talking about life (see the Method section for the assessment of baseline levels). Regression results showed that none of the functions of autobiographical memory had any significant associations with either intimacy or relationship satisfaction (see Tables 3 and 4).

**Table 3:** Regression results for intimacy across studies.

	Study 1		Study 2		Study 3	
	*β*	*p*	*β*	*p*	*β*	*p*
Age	.01	.86	**-.09**	**.05**	-.01	.89
Gender	-.08	.13	-.07	.10	.02	.61
Anxious attachment	**-.15**	**.03**	**-.14**	**.00**	**-.13**	**.01**
Avoidant attachment	**-.55**	**.00**	**-.43**	**.00**	**-.39**	**.00**
Thinking about life	-.06	.34	-.06	.26	-	-
Talking about life	-.05	.38	.02	.73	-	-
Thinking about romantic relationship	-	-	-.02	.65	.01	.83
Talking about romantic relationship with others	-	-	-.06	.23	-.06	.21
Talking about romantic relationship with the partner	-	-	-	-	**.10**	**.04**
TALE Self function	-.04	.51	.02	.76	-	-
TALE Social function	.10	.11	-.12	.06	-	-
TALE Directive function	-.04	.58	.13	.06	-	-
TARE Self function	-	-	.04	.62	.10	.23
TARE Social function	-	-	**.16**	**.03**	-.06	.37
TARE Directive function	-	-	**-.20**	**.01**	-.13	.10
SHARE Self function	-	-	-	-	-.02	.76
SHARE Social function	-	-	-	-	.11	.12
SHARE Directive function	-	-	-	-	.01	.88

*Note*. All values in bold had a p-value lower than .05. TALE = Thinking About Life Experiences Questionnaire. TARE = Thinking About Relationship Experiences Questionnaire (see Study 2). SHARE = Sharing Relationship Experiences (see Study 3).

**Table 4:** Regression results for relationship satisfaction across studies.

	Study 1		Study 2		Study 3	
	*β*	*p*	*β*	*p*	*β*	*p*
Age	**-.10**	**.03**	**-.12**	**.00**	-.06	.11
Gender	-.05	.32	-.03	.43	.01	.81
Anxious attachment	**-.19**	**.00**	**-.18**	**.00**	**-.19**	**.00**
Avoidant attachment	**-.55**	**.00**	**-.47**	**.00**	**-.46**	**.00**
Thinking about life	-.09	.10	-.07	.11	-	-
Talking about life	.06	.27	.05	.31	-	-
Thinking about romantic relationship	-	-	.04	.39	.01	.75
Talking about romantic relationship with others	-	-	.01	.74	.04	.44
Talking about romantic relationship with the partner	-	-	-	-	.04	.34
TALE Self function	-.03	.62	-.04	.59	-	-
TALE Social function	.03	.65	-.07	.23	-	-
TALE Directive function	-.02	.77	.04	.55	-	-
TARE Self function	-	-	.01	.93	.01	.85
TARE Social function	-	-	**.19**	**.01**	.01	.88
TARE Directive function	-	-	**-.19**	**.01**	**-.14**	**.04**
SHARE Self function	-	-	-	-	-.05	.48
SHARE Social function	-	-	-	-	**.17**	**.00**
SHARE Directive function	-	-	-	-	-.04	.56

*Note*. All values in bold had a p-value lower than .05. TALE = Thinking About Life Experiences Questionnaire. TARE = Thinking About Relationship Experiences Questionnaire. SHARE = Sharing Relationship Experiences.

In Study 1 we examined the association between the functions of regular autobiographical memories and relationship quality indicators (i.e., relationship satisfaction and intimacy). Even though the self function was negatively correlated with the relationship quality indicators, the results of the regression analyses did not support our predictions. The functions of autobiographical memories were not associated with relational outcomes.

## Study 2

Whereas Study 1 addressed the association between functions of regular autobiographical memories and romantic relationship quality, in Study 2 we were motivated by the idea that remembering the romantic past may have more direct implications for the quality of one’s relationships. What purposes do the memories about the loved ones serve in the relationship context? While prior work examining this relationship targeted remembering particular incidents in a romantic relationship; such as the first time someone met their spouse [10], here, again, we explore the different functions that the generalized relationship memories serve. Following up on the reasoning in Study 1, if these global assessments consist of conscious processes; that is, individuals are aware of the purposes their romantic memories serve, there may be direct consequences for the relationship quality. For parsimony, we again adopted a self-report methodology, and modified the items in the TALE for assessing the functions of romantic relationship-related memories. Changing the instructions in the original TALE so that the participants answer the items in reference to different classes of memories has also been suggested by the creators of the scale [18]. A secondary aim of Study 2 was to replicate the findings in Study 1, particularly that functions of regular memories were not related to any one of the relationship quality indicators.

Since, in previous work, there is no direct evidence informing us about the link between the self function and romantic relationship quality, we rely on the reported positive associations between the self function and self-esteem [11, 12], and self-esteem and relationship satisfaction [13]. We therefore expect the frequency of self-function to be positively related to relationship quality.

Similarly, we expect the social function to be positively associated with the relationship quality indicators. For instance, if one remembers romantic memories to “coordinate the individual histories of the two partners” [9], intimacy, and relationship satisfaction should increase. In fact, Alea and Vick [10] reported that memories of relationship events with higher qualitative richness -vivid and rehearsed- would correspond to higher marital satisfaction. In a similar vein, warmth and closeness in a relationship increased after recalling a relationship event [7]. Other work with couples has also found that retrieving autobiographical memories about instances where the couple laughed together, as opposed to individual laughter-related events, was related to enhanced marital satisfaction [39].

Finally, we also expect the use of the directive function to be positively associated with relationship quality indicators. Recently, Philippe et al. [15] broadly defined the directive function of memories as having a long-term impact on the cognitions and emotions. They showed that by way of satisfying psychological needs, such as autonomy, relatedness and competence, couple-related memories directively influence relationship satisfaction. In the present context, using romantic relationship memories to guide behavior or to solve current problems should be positively associated with the quality of one’s relationship as it implies actively working on the issues in the relationship.

## Materials and methods

### Participants

Participant recruitment was conducted through the same resource; Prolific. All inclusion criteria and consenting procedures followed Study 1’s lead. Participants in Study 1 were not allowed to participate in Study 2. After excluding 48 participants due to several reasons (e.g., indications of not responding in an honest matter, such as, failure to mark the requested option in the quality check items or not fulfilling the inclusion criteria listed in Study 1), the sample consisted of 410 participants. Sample characteristics are given in Table 1.

### Procedure and measures

All measures were the same as in Study 1 except for a modified version of the TALE [18] which is described below. Participants first received the original TALE then the modified Thinking About Relationship Experiences (TARE) Questionnaire to investigate the functions of memories specific to the current relationship. The administration order of these questionnaires was fixed because we wanted the TALE results to be generalizable to the other studies; that is, responses not to be influenced by prior thoughts about relationships. These are followed by the measures of relationship quality indicators; namely, intimacy and relationship satisfaction. For full descriptions, please see Study 1. This time, we used the full scale for relationship satisfaction.

#### Functions of autobiographical memory

Similar to Study 1, to measure the functions of autobiographical memory, we used the TALE [18]. We again conducted an exploratory factor analysis using varimax rotation and maximum likelihood estimation to investigate the factorial structure of the TALE. Results of the factor analyses, which confirmed the original 3-factor structure, are given in S1 Appendix. For the present study, the five-item scales for self-function, social function, and directive function had good internal reliability levels of .83, .80, and .84, respectively.

#### Functions of relationship-related memories

To measure the functions of relationship-related autobiographical memories, we slightly modified the items of the original TALE Questionnaire to help respondents think back and talk about their relationship. For instance, a directive function item in the original scale being “I think back and talk about my life or certain periods of my life when I want to learn from my past mistakes” was reworded as “I think back and talk about my relationship or certain parts of my relationship when I want to learn from my past mistakes.” For parsimony, we name this version Thinking About Relationship Experiences (TARE) Questionnaire. The participants were warned at the beginning that they were going to answer two similar but slightly different questionnaires; one would be about their life and the other one being about their current romantic relationship. Comparison of all items across two scales are presented in the S1 Appendix section. Again, a 5-point Likert scale (1 = “almost never”, 5 = “very frequently”) was administered in all items. Factor analyses revealed a 3-factor structure as same as the functions in the original TALE (see S1 Appendix), which altogether explained 58.72% of the variance. Internal reliability, Cronbach alpha, levels of five-item scales in the TARE for self-function, social function, and directive function of relationship-related memories (.88, .85, and .88, respectively) were slightly higher compared to the ones in the original TALE.

## Results and discussion

### Descriptive statistics and correlations

Table 5 presents the descriptive statistics of and correlations among study variables in Study 2. Regarding the TALE scale, correlations were very similar to the ones reported in Study 1 in terms of both significance and magnitude. Two differences were the non-significant correlation between self-function and intimacy, and the positive association between social function and anxious attachment.

**Table 5:** Descriptive statistics and correlations among the Study 2 variables.

Variable		*M*	*SD*	1	2	3	4	5	6	7	8	9
1	**TALE Self function**	2.66	0.81	-								
2	**TALE Social function**	2.90	0.77	**.45**	-							
3	**TALE Directive function**	3.26	0.76	**.56**	**.54**	-						
4	**TARE Self function**	2.63	0.87	**.72**	**.46**	**.52**	**-**					
5	**TARE Social function**	2.97	0.84	**.40**	**.69**	**.50**	**.60**	**-**				
6	**TARE Directive function**	3.07	0.86	**.41**	**.50**	**.69**	**.67**	**.67**	**-**			
7	**Intimacy**	5.22	1.39	-.05	-.03	-.04	-.08	.04	**-.10**	-		
8	**Relationship satisfaction**	4.06	0.73	**-.14**	.01	-.09	**-.13**	.08	**-.11**	**.67**	-	
9	**Anxious attachment**	2.48	0.80	**.27**	**.15**	**.20**	**.26**	**.14**	**.20**	**-.32**	**-.38**	-
10	**Avoidant attachment**	2.18	0.70	**.13**	-.05	.06	**.13**	-.08	.05	**-.50**	**-.60**	**.42**

*Note*. All values in bold had a p-value lower than .05. TALE = Thinking About Life Experiences Questionnaire. TARE = Thinking About Relationship Experiences Questionnaire.

Correlations between the same functions in the TALE and TARE ranged between .69 and .72, meaning that they overlap with each other to some extent but are not the same constructs. Significant correlations between functions in the TARE and relationship quality variables showed that self-function in the TARE was negatively linked to relationship satisfaction while directive function in the TARE is negatively associated with both intimacy and relationship satisfaction.

### Regression results

Regression results with the control variables revealed that, similar to the Study 1 results, there was no link between functions of memory in the original TALE scale and neither relationship satisfaction nor intimacy (see Tables 3 and 4). Both the intimacy and relationship satisfaction had positive and negative links with social and directive functions in the TARE respectively. These effects were significant although we controlled for the effects of confounding variables including the attachment types (see Tables 3 and 4). Effect sizes were small (*f*^2^ = .01 for the effect of social function on intimacy, *f*^2^ = .02 for the effect of directive function on intimacy as well as for the effects of social and directive functions on relationship satisfaction).

In our regression analysis, similar to the findings in Study 1, there was no link between functions of memory in the original TALE scale and neither relationship satisfaction nor intimacy (see Tables 3 and 4). In turn, consistent with our predictions, functions measured by TARE had different associations with the relationship quality indicators. Even after controlling for attachment, age and gender, social function was positively associated with intimacy and relationship satisfaction; whereas directive function was negatively related to them. This finding supports the idea that relationship-related memories are used in individuals’ daily lives and are related to relational outcomes. This pattern contributes to the literature in that not only singled out episodes in relationships, such as vacation with the partner [7] or first time someone met their spouse [10] would function to increase intimacy levels or satisfaction in a relationship but also generalized evaluations of multiple episodes, such as the items in TARE, have associations with the quality of one’s relationship. Overall, the present study was a first in showing that the functions of relationship-related memories, when studied together, are related to the relationship quality. This association is qualitatively different from the pattern with regular, non-relationship-themed, memories which is a finding points to the need for distinguishing different themes/classes of memories when examining their functions.

## Study 3

It has been established that memory sharing is one of the primary functions of autobiographical memory [40]. Sharing autobiographical memories has been shown to lead to relationship closeness across cultural settings [41]. It is, therefore, a critical omission in the memory function literature that the role of different interlocutors is rarely considered. Given that the functions and characteristics of the shared vs non-shared memories differ [e.g., 42], events shared with an intimate other are likely to differ in function from events shared with other people [22]. Therefore, the main aim was to explore whether functions of relationship-related events were similarly associated with relationship quality indicators when they were shared with the romantic partner vs any other person. It is, for instance, possible that when shared memories have a tendency to serve a self function, the quality of the relationship may deteriorate due to the realization of the partner of not being included in the meaning-making process of the romantic experience. Thus, associations between memory functions and relationship quality may be different than when they were not shared with the partner or in some cases, such as when the social function is involved, may be enhanced.

## Materials and methods

### Participants

We followed the same procedure explained in the first two studies and recruited participants through Prolific. Participants in the first two studies were not allowed to participate in this study. There were 455 participants in the dataset after the exclusion of 63 participants because of various reasons such as failure in quality check questions. Characteristics of the final sample are given in Table 1.

### Procedure and measures

#### Functions of relationship-related memories

We used the TARE (Thinking about Relationship Experiences) again to examine whether we can replicate our findings in Study 2. Furthermore, we adapted the items in the TARE to investigate the functions of relationship memories when they were shared with the current partner. The modified scale is referred to as SHARE (Sharing Relationship Experiences) from here on. As an example for the difference between the TARE and SHARE scales, the TARE item “I think back and talk to other people about my relationship or certain parts of my relationship when I want to learn from my past mistakes” was used as “I think back and talk to my partner about my relationship or certain parts of my relationship when I want to learn from my past mistakes” in the SHARE. Comparison of items across scales are presented in the S1 Appendix section.

Exploratory factor analysis revealed a 3-factor structure for the TARE in this study too with an explained variance of 56.72% in total (see S1 Appendix). Although two items loaded similarly onto two different factors, the results were almost identical when those items were excluded except that the effect of TARE directive function on relationship satisfaction in Table 4 became marginal (*β* = -.12, *p* = .07). Thus, we continued with the original 3-factor structure. Five-item scales for self-function, social function, and directive function in the TARE had good internal reliability levels of .87, .83, and .86, respectively.

The SHARE had a 2-factor structure in the exploratory factor analysis with an explained variance of 58.26% (see S1 Appendix). The first factor was again representing the self function with the same 5 items. Social and directive functions however, overlapped and constituted a separate function together. This indicates that talking about the relationship problems with the partner to guide future behaviors for example (i.e., directive function) also has a role in bonding the partners with each other (i.e., social function). For the present results to be comparable with the findings using both the TALE and TARE as well as considering the theoretical differences between social and directive functions, we still used these two factors of SHARE separately in our analysis. The internal reliability levels were also supporting our decision to use the three functions separately. Five-item scales for self function, social function, and directive function had good internal reliability levels (Cronbach alphas) of .91, .89, and .85, respectively.

#### Other variables

For assessing relationship quality and attachment styles, we used the same measures in previous studies. Internal reliability levels for relationship satisfaction, anxious attachment, and avoidant attachment were .92, .93, and .95 respectively.

## Results and discussion

### Descriptive statistics and correlations

Descriptive statistics of and correlations among study variables in Study 3 are presented in Table 6. Correlations between functions in the TARE and relationship quality variables showed negative association of self function and directive function with relationship satisfaction. All three functions were positively linked to anxious attachment. Avoidant attachment was positively linked to self function, but negatively linked to social function.

**Table 6:** Descriptive statistics and correlations among the Study 3 variables.

Variable		*M*	*SD*	1	2	3	4	5	6	7	8	9
1	**TARE Self function**	2.26	0.86	-								
2	**TARE Social function**	2.75	0.84	**0.54**	-							
3	**TARE Directive function**	2.89	0.85	**0.66**	**0.67**	-						
4	**SHARE Self function**	2.43	0.93	**0.73**	**0.40**	**0.52**	-					
5	**SHARE Social function**	3.40	0.90	**0.30**	**0.52**	**0.48**	**0.48**	-				
6	**SHARE Directive function**	3.09	0.84	**0.40**	**0.49**	**0.66**	**0.63**	**0.72**	-			
7	**Intimacy**	5.22	1.47	-0.09	-0.03	-0.08	0.00	**0.17**	0.07	-		
8	**Relationship satisfaction**	4.03	0.79	**-0.16**	0.02	**-0.10**	-0.07	**0.22**	0.05	**0.66**	-	
9	**Anxious attachment**	2.43	0.82	**0.29**	**0.17**	**0.21**	**0.23**	**0.12**	**0.16**	**-0.32**	**-0.41**	-
10	**Avoidant attachment**	2.11	0.72	**0.11**	**-0.09**	0.00	-0.02	**-0.28**	**-0.16**	**-0.48**	**-0.62**	**0.46**

*Note*. All values in bold had a p-value lower than .05. TARE = Thinking About Relationship Experiences Questionnaire. SHARE = Sharing Relationship Experiences.

Correlations between the same functions in the TARE and SHARE ranged between .52 and .73, which showed that they somewhat overlap with each other but tap into different constructs. Correlations between functions in the SHARE and relationship quality revealed that only social function had significant associations with intimacy and relationship satisfaction.

### Regression results

Regression results in Table 3 showed that none of the functions either in TARE or SHARE had significant associations with intimacy. Results about relationship satisfaction however, showed that directive function in the TARE and social function in the SHARE were negatively and positively associated with relationship satisfaction, respectively. In the model with the control variables including attachment (Table 4), effect sizes were relatively small: *f*^2^ = .01 for the effect of TARE directive function on relationship satisfaction, and *f*^2^ = .02 for the effect of SHARE social function on relationship satisfaction.

Furthermore, as described in S1 Appendix, we also conducted the same analysis using the 2-factor structure of the SHARE (i.e., social function and the combined factor of social and directive functions). The results with the 2-factor structure of the SHARE revealed that the negative effect of TARE directive function on intimacy was in line with the finding in Study 2 (see Table 3). This effect was not significant in Study 3 when the 3-factor structure was used (see Table 3). The combination of the social and directive functions in the SHARE had a positive effect on relationship satisfaction. This effect was in line with the positive effect of social function in Study 3 when the 3-factor structure was used (see Table 4). SHARE directive function alone was not significant (see Table 4). We also explored whether the associations of memory functions with relationship quality depended on relationship duration across the three studies (30 interactions in total). The results showed that only one out of 30 interactions was noteworthy (see S1 Appendix for details). The association of the SHARE social function with intimacy depended on relationship duration (*b* = .35, *p* < .001). Simple slope analyses showed that the SHARE social function had a significant positive association with intimacy in people with longer relationship duration (1 SD above the sample mean; *b* = .56, *p* < .001), but no association in people with shorter relationship duration (1 SD below the sample mean; *b* = -.13, *p* = .30). Hence, our exploratory examinations did not reveal a strong role of relationship duration in the associations between memory functions and relationship quality.

Thus, when the addressee of the memory sharing activity was defined as the romantic partner, the way functions were linked to the quality of relationships slightly differ from when sharing with the partner is not specified. The positive association between the social function and relationship satisfaction (see TARE results in Study 2) is still intact however the negative link between the use of directive function and satisfaction (see TARE results in Study 2 and Study 3) is not there anymore. This is the first study that we know of to show that the associations between the intended reasons to share romantic memories and relationship quality may slightly change when the romantic partner is the interlocutor. The implications of this finding are discussed further below.

## General discussion

In the present study we aimed to examine the association of functional use of memory and romantic relationship quality in three studies. We did so by focusing on generalized views on memories in order to examine the three overarching functions together. We first looked at whether everyday memories’ functions and relationship quality were linked. In a second study, we shifted our focus to the functions relationship-related memories serve; and in the third study, we specified the interlocutor as the romantic partner when considering functions of romantic memory sharing and relationship quality association.

The predicted positive relations between the three functions and functions of everyday memories were not confirmed; none of the functions were associated with the quality indicators. We did, however, observe the functions of romantic memories to be associated with romantic relationships outcomes. How shall these findings inform current theorizing regarding functional remembering in social context?

The lack of association between reasons to remember everyday autobiographical events and the quality of one’s relationships is in contrast with the previous findings showing that functional remembering -all three functions- is related to having positive relationships [11]. A closer look, however, reveals that the association in the Waters study was reported for single events only but not for recurring or general events. A summary of one’s lifetime periods, as indexed by the TALE, may not have the functional power to have an immediate effect on the relationship quality but a relationship-specific single event, such as “when we saw that movie together” or even general events such as “our walks to school together” might have a binding role for other relationship events; and therefore, its functional relations to the quality of romantic relationships may be more salient. The present findings therefore suggest that the general tendency with which individuals remember their past life may not be associated with the quality of their romantic relationships.

Alternatively, the items in the two studies may have tapped different aspects of the so-called self function. While Waters [11] used the Centrality of Event Scale which measures whether the event recalled constitutes a key part of one’s identity; therefore, taps the identity aspect; the TALE scale in the present study examines the continuity of the self in time; therefore, coherence of a self-concept. Pillemer [8] has noted that conceptual categories that the TALE measures may not encompass the full spectrum of the subfunctions of a particular category. Therefore, self function as indexed by the TALE items may not be associated with the relationship quality but whether or not a particular event helps defining the self is. Further research with a focus on the spectrum of the subfunctions is required to support this interpretation.

With regards to the romantic memories (Study 2), results suggest that the social function was positively related to both relationship satisfaction and intimacy; whereas directive function was found to be negatively correlated with both of them. The positive association between the social function and how it is related to relationship satisfaction [10] and intimacy [7] has been shown previously with specific, one-time relationship memories, such as first-sight or first-kiss. The novelty of the present findings is that across different settings–as the items in the scale imply- social function is similarly related to the relationship outcomes.

An unexpected finding was the directive function to be negatively associated with relationship quality when relationship memories are shared with other people. Previous research has tracked the influence of a single episode; a specific memory’s directive power and found that it was positively associated with one’s satisfaction in a relationship [8, 15]. A relationship-related memory; for instance, a prior quarrel, could naturally be used constructively within the relationship for problem-solving purposes or to fine-tune particulars of future behavior. When faced with a problem, individuals would bring to mind memories of situations involving a similar problem and use that particular memory to work through the challenge [1, 17, 43]. This guidance would reflect positively on relationship satisfaction. In the present study, however, we were dealing with a global evaluation of how frequently relationship memories are used for directive purposes. If one’s perception of the frequency of the use of memories for problem-solving purposes, is high, it might indicate that the frequency of the problems to be solved is also high. Following that logic, if the perception of the number of problems (that needs to be solved) in a relationship is high, it is highly likely that the perceived satisfaction in the relationships would not benefit it.

The negative effect of directive function on relationship quality vanishes when relationship memories are shared with the partner rather than anonymous others (Study 3). This is further support for the idea that memories are used based on the changing dynamics of the situation or context [17, 22]. Why is sharing relationship memories for directive purposes with others detrimental for relationships? Previous research showed that discussing relationship problems with friends harms relationship quality, if similar discussions do not take place with the partner [44]. Perhaps the discussion with the partner brings the opportunity to take the perspective of the partner and smoothly resolve the conflicts, which is not possible when relationship memories are told to others.

Differential results across social and directive functions may also be due to the valence of the memories. Previous research showed stronger associations of social and directive functions with positive and negative memories, respectively [20]. Thus, our findings (in Study 2) revealing the beneficial effect of social function, but detrimental effect of directive function on relationship quality may not be surprising. Linking this finding with the relationship research, perhaps social function is more salient in capitalization attempts (i.e., sharing good news with others/partner; e.g., [45]), whereas directive function is more salient during discussions about relationship problems [44]. These questions await future research.

An unexpected finding was the lack of social function’s effect on intimacy for the relationship memories shared with the partner despite its positive effect on relationship satisfaction. Previous studies showed the bonding roles of disclosure and capitalization in romantic relationships [45, 46]. One possible explanation of not observing the positive trend here is that we did not consider reaction of the partner. Future research should examine whether social function builds intimacy when the partner shows constructive and supportive responses [47].

All in all, it was critical to show that global evaluations of memory functions also have direct associations with the quality of the relationships. Further, as we noted previously, here we operationalize function as the deliberate use of the memories [1]. Prior work focusing on singled out relationship episodes, in turn, rely on non-conscious uses of memories. Since it has been suggested that some functions may be less accessible than others [22], future work is needed to make this distinction clearer. For instance, the present studies could be replicated with measures other than self-reported uses. One way is to conduct content analysis of functional use on the memory narratives [e.g., 48].

The present study further shows that memory functions in the modified versions of the TALE, which we called the TARE (Thinking about relationship experiences) and SHARE (Sharing relationship experiences with the partner) were differentially linked to relationship quality. We conclude that TARE and SHARE have utility as separate tools to examine reminiscence dynamics in the romantic relationship context, and to inform intervention or counseling programs. A cautionary note, however, is that the present design employed a fixed ordering of the questionnaires which may have influenced the participants’ responses. Even though there was a clear warning in the instructions that there would be two very similar questionnaires to fill out, there is no way to know whether responding to the TALE (or the TARE, in the second study) questions had an alerting or inhibiting role on the subsequent scales. Since our main aim in this research was to examine the roles of memory functions by comparing our results using the original measure (TALE) and modified measures (TARE and SHARE) with each other, in none of our studies the TARE or SHARE were used separately. Future studies planning to use these measures should take this into account and test the replicability of our findings in contexts where the TARE or the SHARE are used in isolation.

The three-function model is suggested to be used as a conceptual model [8] with heuristic utility [17] for understanding functional uses of the memories broadly. In fact, each function is regarded as an organizatory unit to include many subpurposes [22]. Targeted experimental manipulations are needed to fine-tune each function’s association to relational outcomes. With the present design, it may not be entirely possible to rule out alternative explanations; such as, individuals that are highly satisfied in their relationships may tend to remember for social reasons or individuals who do not possess feelings of intimacy in the relationship may tend to use memories mostly for problem-solving purposes. Future research should also identify whether positive or negative relationship memories function the same way. Some newly identified functions, such as the mood-enhancement function, are very loosely captured by the three categories [49] but would be very relevant to examine for the romantic relationship context.

Our operationalization of the romantic relationship quality (satisfaction and intimacy) should be considered as a first step in exploring the wide range of possible qualities [e.g., 26]. Exploring conflict, for instance, would be interesting in terms of how memory is used to deal with negative relational outcomes. Future research should also consider the moderating roles of other relationship characteristics (e.g., married vs. cohabitating, same-sex vs. heterosexual, monogamous vs. non-monogamous relationships).

It should also be noted that psychological well-being has been previously associated with the memory functions [11]. Therefore, it is possible that our memories’ influence on one’s relationship quality might be through their effects on general wellbeing and not because of their direct effects on relationships. It would be worthwhile for the future studies to focus on the mediating effects of psychological health on this mechanism.

In conclusion, our findings suggest that when remembering has consequences in terms of the quality of a romantic relationship, how memories are used change depending on their theme (relationship-related or not) and who the social partner is (romantic other or not). Future studies should consider fine-tuning these broad functional categories in order to further understand the causal mechanisms. For instance, whether or not remembering a particular relationship incident directively might hinder relationship satisfaction. Together, the extant findings suggest adopting a contextual approach as they revealed not only that functions relate to different social contexts such as romantic relationships but also that they consider the role of the social partner.

## Supporting information

S1 Appendix (XLSX)
